# Correlation between Serological Biomarkers and Disease Activity in Patients with Inflammatory Bowel Disease

**DOI:** 10.1155/2019/6517549

**Published:** 2019-12-25

**Authors:** Mengque Xu, Mengsha Cen, Xiaoli Chen, Haotian Chen, Xing Liu, Qian Cao

**Affiliations:** ^1^Department of Gastroenterology, Sir Run Run Shaw Hospital, School of Medicine, Zhejiang University, Hangzhou, China; ^2^Department of Medical Records and Statistics, Sir Run Run Shaw Hospital, School of Medicine, Zhejiang University, Hangzhou, China

## Abstract

**Background:**

Current biomarkers have been routinely used noninvasive methods for assessing disease activity of inflammatory bowel disease (IBD), but none of them are specific. This study was aimed to determine the performance of the serological biomarkers for detecting disease activity in patients with IBD.

**Methods:**

A prospective study that included 73 ulcerative disease (UC) subjects, 141 Crohn's disease (CD) subjects, and 30 of them complicated with *C. difficile* infection (CDI) were diagnosed at a single-institution IBD center. Disease activity was assessed using by Truelove and Witts criteria for UC and Harvey Bradshaw Simple Index for CD. Serological inflammatory biomarkers were compared in different severity groups. Receiver operator curve analyses assessed the performance of each biomarker in discriminating disease states.

**Results:**

For UC patients, elevated monocyte counts, C-reactive protein (CRP), and decreased lymphocyte counts and lymphocyte/monocyte ratio (LMR) significantly differed between subjects with active and inactive UC. LMR of 3.1 was 76% sensitive and had a specificity of 67% for active UC. For CD patients, higher values of neutrophils, monocytes, neutrophil/lymphocyte ratio, CRP, fibrinogen, and lower values of LMR and hemoglobin were significantly different between subjects with active and inactive CD. None of the biomarkers included had a good correlation with disease activity (area under the ROC Curve < 0.70).

**Conclusions:**

A low LMR represents an inexpensive, readily available test with a promising value to identify disease activity in UC patients, whereas none of the inflammatory biomarkers showed a discriminative value in disease activity of CD.

## 1. Introduction

Inflammatory bowel disease (IBD), with its high incidence and prevalence, is now regarded as a worldwide healthcare issue [1–3]. Crohn's disease (CD) and ulcerative colitis (UC), the two major types of IBD, are chronic relapsing immunologic disorders of the bowel, and they appear to result from dysregulation of the immune system [4]. The assessment of IBD disease activity helps to guide clinical decisions of subsequent therapy [5]. Biomarkers in IBD can aid in the monitoring of disease severity in clinical practice, including erythrocyte sedimentation rate (ESR) and C-reactive protein (CRP) [6–8]. However, given the suboptimal performance of currently available biomarkers, endoscopy with biopsies remains the gold standard for evaluating and monitoring the inflammatory activity, but limited in use owing to its invasiveness and the need for on-demand specimen collection [9]. Thus, the search for easily accessible and cost-effective biomarkers that can be used to assess the disease activity is necessary and urgent for optimal management of IBD.

Recently, various serum markers, including the leukocyte differentials and neutrophil/lymphocyte ratio (NLR) have been evaluated as surrogate markers for predictive and prognostic values in various entities, such as rheumatoid arthritis, pancreatitis, and several malignancies [10–13]. Recent research focusing on cost-effective biomarkers in IBD has reinvigorated the examination of white blood cell patterns, and a few studies have showed the potential of neutrophil and lymphocyte counts and their ratio to assess the disease activity either in UC or CD [14, 15]. Blood mononuclear cells were also found to correlate with UC activity [16]. However, the heterogeneity of the study populations led to variation, and there is a paucity of data regarding the utilities of other cell types and ratios reported, especially in CD patients. In this study, we aimed to elucidate the association of leukocyte values and the ratios with IBD activity and also differentiate it from *Clostridium difficile* infection (CDI).

## 2. Methods

### 2.1. Study Subjects

The prospective study sample comprised 214 initially diagnosed IBD patients at the IBD unit from a single hospital between March 2017 and February 2018, including 73 UC and 141 CD patients, 30 of them with *C. difficile* infection (CDI). The diagnosis of UC and CD was separately based on standard clinical, radiological, endoscopic, and histological criteria. Informed consent was granted from all the patients, and ethical committee approval was obtained from the Ethics Committee of the Sir Run Run Shaw Hospital, Zhejiang University.

Data including patients' age at diagnosis, gender, and localization of the disease were retrieved from the medical database. Information of the patients' laboratory studies, endoscopic reports, and pathology reports at diagnosis was also recorded. The ratios of leukocyte fractions including neutrophil-to-lymphocyte ratio (NLR), lymphocyte-to-monocyte ratio (LMR), and neutrophil-to-monocyte ratio (NMR) were calculated. Patients were stratified according to the disease duration.

Exclusion criteria included (1) prior treatment with corticosteroids, hematological, neoplastic disorders, intestinal tuberculosis, or coagulopathy, (2) clinical evidence of active infection (except CDI), such as positive urine and blood cultures, documented skin infection, infiltrates on chest x-ray, and so on, and (3) patients <15 years of age.

### 2.2. Disease Activity

The UC disease activity was assessed by Truelove and Witts criteria [5, 17], as the criteria allow a simple and rapid stratification of UC patients and have been validated for over 60 years. To evaluate the UC disease activity index, the Mayo scoring system was used as previously described [18]. Patients with UC were classified as mild, moderate, or severe based on the number bloody stools per day, body temperature, pulse, hemoglobin, and ESR. Active UC was considered as having moderate or severe disease, whereas the remission period was defined as the mild group. For CD, the disease activity was classified by the Harvey Bradshaw Simple Index [19], based on the five variables (general well-being, severity of abdominal pain, number of liquid stools daily, presence of abdominal mass, and complications). Patients categorized as having an HBI score >4 were accepted as having active CD, whereas patients having an HBI score ≤4 were considered to be in the remission group.

### 2.3. Statistical Analysis

Continuous variables with normal distribution were presented as mean ± standard deviation or, in the case of nonnormally distributed data, as median (range). Comparisons of proportion between different groups of patients were analyzed using the Chi-squared test. All normally-distributed values were carried out using student's *t*-test. Multivariate logistic regression analysis was used to evaluate the ratios (OR) and 95% confidence intervals (CI) for disease activity with significant findings in univariate analysis. Spearman's rank correlation coefficient was used to assess the correlations between the disease activity index of the disease and laboratory parameters (NLR, LMR, and NMR). Receiver operating characteristic (ROC) curve analysis was used to assess the accuracy of each biomarker (area under the curve, AUC) and identify optimal cutoff values of NLR, LMR, and NMR with maximum sensitivity and specificity for differentiation of activation of UC or CD from remission. A *P* value of less than 0.05 was considered statistically significant. The Statistical Package for Social Sciences (SPSS) 22 (Chicago, Illinois, USA) was used to analyze the data.

## 3. Results

A total of 214 patients diagnosed with IBD were included, 73 (34.1%) with UC and 141 (65.9%) with CD. One-hundred and forty-six (68.2%) were male, and the mean age of the whole cohort was 36 years (range 15–73 years). The demographic and disease characteristics of the patients included in the study are summarized in Table 1.

In the UC group, the mean age of the group was 43.4 (18–73 years), and 42 (57.5%) were male. Eighteen (24.7%) patients were complicated with CDI, and the left 55 patients without CDI, 18 (24.7%) of whom were deemed as remission group, and 37 (50.7%) were classified into active group. Four (5.5%) patients with UC had a proctitis, 24 (32.9%) had left-side colitis, and 45 (61.6%) had pancolitis. In the CD group, the mean age of the group was 32 years (15–62 years), and 104 (73.8%) were male. Except for 12 (8.5%) had CDI, among the left 129 (91.5%) CD patients, 44 (31.2%) were categorized as the active group. One-hundred and seven (75.9%) patients had lesions involved with colon.

### 3.1. Serological Biomarkers for Diagnosis and Disease Activity

When comparing the mean values of the serological markers between patients with and without disease activity, we found values of lymphocytes (1.4 (0.6) vs. 1.8 (0.7), *P*=0.040), monocytes (0.9 (0.5) vs. 0.6 (0.3), *P*=0.023), LMR (2.2 (1.6) vs. 3.5 (1.7), *P*=0.011), and CRP (34.2 (40.3) vs. 12.2 (29.0), *P*=0.043) had statistically significant differences between UC patients with and without disease activity. Inflammatory markers, such as CRP (37.1 (39.0) vs. 12.2 (29.0), *P*=0.037) and fibrinogen (4.0 (1.0) vs. 3.2 (1.1), *P*=0.035, were found to be significantly elevated in UC with CDI compared with inactive UC without CDI patients (Table 2). Further multivariate analysis showed a significantly lower level of LMR which was observed in the active UC patients without CDI than inactive UC patients without CDI (OR = 0.650; 95% CI: 0.457–0.925; *P* < 0.05).

By contrast, compared with the CD patients in remission period, in active CD patients, serological markers had higher mean values, being these differences statistically significant for WBC (8.7 (3.2) vs. 6.5 (2.1), *P*=0.000), neutrophils (6.3 (2.8) vs. 4.5 (1.7), *P*=0.000), monocytes (8.7 (3.2) vs. 6.5 (2.1), *P*=0.000), NLR (5.5 (4.5) vs. 4.0 (2.0), *P*=0.010), CRP (47.4 (47.2) vs. 19.3 (21.8), *P*=0.010), and fibrinogen (4.8 (1.3) vs. 4.3 (1.2), *P*=0.018), while had lower mean values of LMR (2.3 (1.2) vs. 3.1 (1.9), *P*=0.009) and Hb (11.1 (2.3) vs 11.9 (1.9), *P*=0.047). CD with CDI patients compared with CD patients in remission showed significantly higher CRP (35.6 (22.8) vs. 19.3 (21.8), *P*=0.018) (Table 3). Further multivariate analysis showed a significantly higher level of serum neutrophils, and CRP was observed in the active CD patients than inactive CD patients without CDI (OR = 1.395; 95% CI: 1.134–1.717; *P* < 0.05; OR = 1.021, 95% CI: 1.006–1.036; *P* < 0.05).

### 3.2. Correlation of Serological Biomarkers and Disease Activity Index

**Figure 1:**
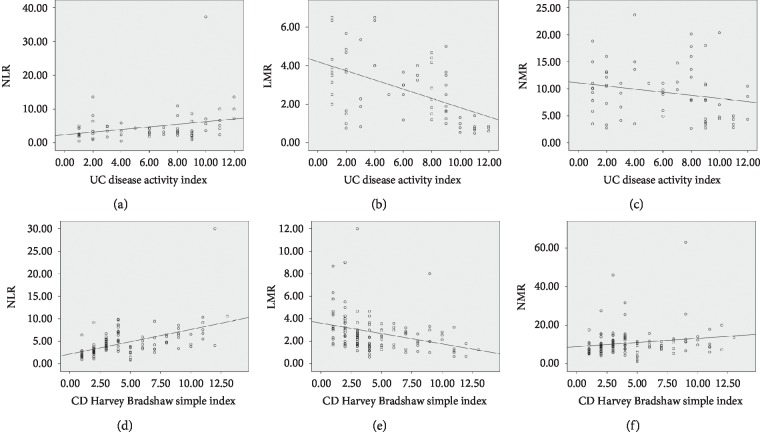
The correlations between the disease activity index of ulcerative colitis (UC)/Crohn's disease (CD) and laboratory blood cell ratios.

We then assessed correlation coefficient between serological biomarkers (NLR, LMR, and NMR) and disease activity index of UC/CD (Figure 1). Overall, in UC patients without CDI, serum NLR showed a positive correlation with the disease activity index (*r* = 0.321; *P* < 0.01), while LMR and NMR inversely correlated with the disease activity index (*r* = −0.55, *P* < 0.001; *r* = −0.26, *P* < 0.05). In CD patients without CDI, LMR and NMR showed a positive correlation with HBI (LMR, *r* = 0.579, *P* < 0.001; NMR, *r* = 0.224, *P* < 0.05), while LMR was inversely correlated with HBI (*r* = −0.418, *P* < 0.001).

### 3.3. Diagnostic Biomarker Performance

**Figure 2:**
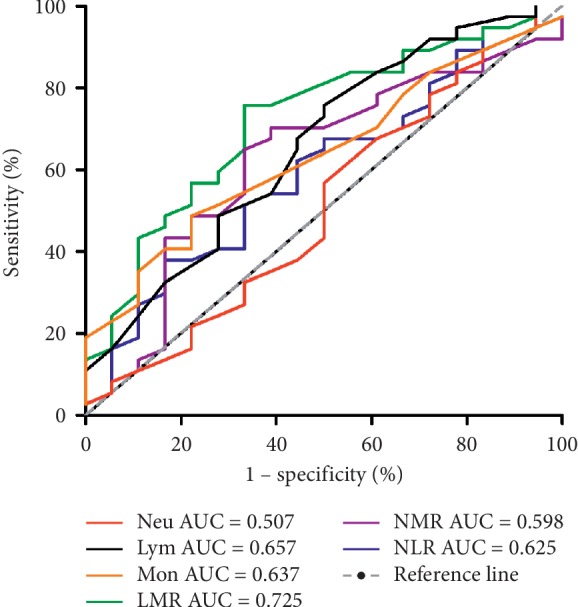
Receiver operating characteristic (ROC) curve of various leukocyte subtypes and ratios in predicting active disease for ulcerative colitis (UC).

**Figure 3:**
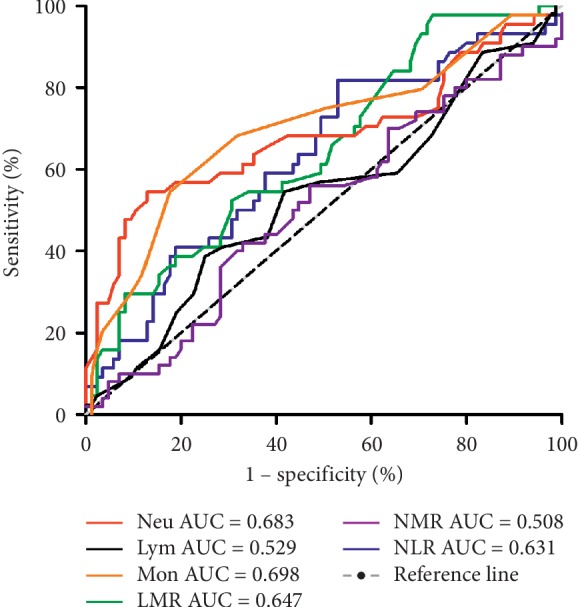
Receiver operating characteristic (ROC) curve of various leukocyte subtypes and ratios in predicting active disease for Crohn's disease (CD).

We identified several leukocyte ratio markers that were able to differentiate active UC/CD from UC/CD in the remission period, respectively. ROC analyses revealed that LMR (AUC = 0.722, 95% CI, 0.580–0.863) ratio was the best biomarker to differentiate active UC from UC remission patients. An LMR cutoff value of 3.1 had a sensitivity of 76% and specificity of 67% with lower values becoming progressively more specific (Figure 2). The diagnostic accuracy of the serological biomarkers in CD patients was bad, as none of them had an AUC >0.7 (Figure 3).

## 4. Discussion

Recent studies have confirmed fecal calprotectin is the best biomarker for evaluating disease activity in IBD patients [20, 21]. However, it is limited in clinical practice owing to its cost and discommodious sample collecting and processing. In the present study, we evaluated the diagnostic accuracy of serological biomarkers to determine the disease activity in UC and CD patients, as the biomarkers were universally monitored at routine clinical practice and thus easily accessible.

CRP and ESR are the most routine-used inflammatory indices for determining disease activity in patients with IBD [14]. However, the results of previous studies were disappointing, owing to the two biomarkers (CRP and ESR) with low sensitivity and specificity for reflecting the bowel inflammation [22, 23]. Previous studies have shown hypercoagulable state was associated with intestinal inflammation state, and serum fibrinogen level was correlated with the severity of the acute phase response [24, 25]. A decreased serum albumin level has also been described to associate with increased systemic inflammatory load [26]. In this respect, in addition to the serum white blood differentials, we also investigated the biomarkers mentioned above.

In the current study, we found a significant association between elevated monocytes and CRP in patients with active UC compared with those with inactive UC, as well as decreased lymphocytes and LMR in patients with active UC, while further multivariate analysis showed a significant lower LMR was found in patients with active UC. Although there were more biomarkers, including the neutrophils, monocytes, HB, CRP, ESR, fibrinogen, NLR, and LMR, which were described associated with the disease activity of CD, only higher neutrophils and CRP were found in patients with active CD if assessed by multivariate analysis. The diagnostic accuracy of the serological biomarkers in CD patients was disappointing, as none of them had an AUC >0.7.

To get the more accurate results, we excluded the patients who had got medications (corticosteroid, thiopurines, etc) before the study, taking into account the potential influences that medications could have on the outcomes. Besides, infections are also confounders that can affect the leukocyte differential counts. Therefore, CDI, one of the active infections, was also examined in this study. We found elevated CRP and fibrinogen levels were associated with UC patients with CDI, comparing with inactive UC patients. Also, CRP levels in CD patients with CDI were statistically significant and higher than inactive CD patients.

The neutrophils play a key role in the active inflammatory response and are proposed to contribute to the destructive tissue cascades by secretion of interleukin-1, interleukin-6, and tumor necrosis factor-*α*. Moreover, previous studies in patients with IBD have strongly revealed that their lymphocyte function is abnormal at both the peripheral and mucosal level [27]. NLR was first identified as a parameter of systemic inflammation in 2001 [28], and it has been extensively reported thereafter in both malignant and nonmalignant conditions. Previous studies described NLR was a controversial marker in IBD. Elevated NLR was found in patients with active UC in comparison with healthy controls [14, 29]. However, two recent studies showed NLR was effective in differentiating active UC/CD from healthy controls, but not from inactive UC/CD [15, 16]. Through this study, we also did not find the diagnostic value of NLR in the disease activity of either UC or CD.

Factors such as medications can influence the leukocyte-type counts. We know steroids can increase neutrophil count and subsequently the NLR. In this study, as mentioned above, we accounted for the potential confounders by enrolling only the initially diagnosed IBD patients, and other leukocyte subtypes and ratios were examined. Monocytes, a subset of leukocytes, differentiate into macrophages and dendritic cells in the inflamed tissues, involving in innate immunity by releasing proinflammatory cytokines, chemokines, and pathogen-associated molecular patterns [30–32]. Thus, activation of monocytes is prospected to be initiated during the active phase of IBD. Cherfane et al. performed a retrospective study in UC patients and reported the monocytes and LMR were promising biomarkers in UC. Similarly, our data revealed a significant association between monocytosis and disease activity of IBD, as well as the LMR. Besides, LMR had the best AUC (0.722) in UC patients. An LMR value lower than 3.1 carries a 76% sensitivity value for active UC. However, its diagnostic accuracy in differentiating disease activity of CD was undesirable. Our findings can be explained by the role that monocytes and lymphocytes play in the innate immune responses in such an inflammatory disease as IBD.

There are several strengths for this study. Firstly, it is a prospective study to prove the utilities of inflammatory biomarkers for severity stratification in patients with UC and CD. To our knowledge, there are limited data for analyzing the efficacy of these biomarkers in CD. Secondly, the study cohort is homogenous, in which the diagnosis and severity disease evaluation were allocated based on standardized definitions. Additional strengths of our study were inclusion of active disease, quiescent disease, CDI, and taking into account the influence of medications. However, this was a single-center cohort with a relatively small sample size.

In conclusion, among the inflammatory biomarkers, including CRP, ESR, fibrinogen, leukocytes, NLR, LMR, and NMR, the LMR has the highest discriminatory capacity for severe UC, with an optimal cutoff value of 3.1, but none of them with a discriminative value in evaluating disease activity of CD. Further work with multicenter studies to assess the biomarkers is warranted.

## Figures and Tables

**Table 1:** Characteristics of the inflammatory bowel disease cohort (*N* = 214).

Variables	UC cohort	CD cohort
Total number	73	141
With CDI, *n* (%)	18 (24.7)	12 (8.5)
Age (years), median (range)	43.4 (43) [18–73]	32 (31) [15–62]
Male gender, *n* (%)	42 (57.5)	104 (73.8)
BMI (kg/m2) $\left(\bar{x}\pm S\right)$	20.8 (2.9)	18.6 (2.6)
Disease activity		
Without CDI		
Inactive/active	18 (24.7)/37 (50.7)	85 (60.3)/44 (31.2)
With CDI		
Inactive/active	2 (2.7)/16 (21.9)	6 (4.25)/6 (4.25)
Localization of disease		
Without colitis/with colitis		34 (24.1)/107 (75.9)
Distal colitis/left-sided/pancolitis	4 (5.5)/24 (32.9)/45 (61.6)	

UC, ulcerative colitis; CD, Crohn's disease; CDI, *Clostridium difficile* infection; BMI, body mass index.

**Table 2:** Demography and basic laboratory values of ulcerative colitis patients.

Variables	UC without CDI, inactive	UC without CDI, active	^*∗*^ *P* value	UC with CDI	^*∗*^ *P* value
Total number (%)	18 (24.7)	37 (50.7)		18 (24.7)	
Epidemiology					
Age (years), median (range)	43.0 (25–58)	43.0 (18–73)	0.580	41.0 (24–69)	0.823
Male gender (%)	8 (44.4)	21 (56.8)	0.391	13 (72.2)	0.091
BMI (kg/m^2^) (SD)	21.1 (3.0)	20.1 (2.3)	0.171	21.7 (3.6)	0.625
Laboratory examination					
WBC (10^9^/L) (SD)	8.6 (3.9)	8.7 (4.2)	0.923	11.6 (11.6)	0.294
Neutrophil (10^9^/L) (SD)	6.0 (3.5)	6.2 (4.0)	0.819	6.3 (1.8)	0.788
Lymphocyte (10^9^/L) (SD)	1.8 (0.7)	1.4 (0.6)	0.040	1.6 (0.6)	0.404
Monocyte (10^9^/L) (SD)	0.6 (0.3)	0.9 (0.5)	0.023	0.9 (0.6)	0.157
NLR	3.8 (3.09)	5.5 (6.2)	0.273	4.2 (1.3)	0.622
LMR	3.5 (1.7)	2.2 (1.6)	0.011	2.5 (1.3)	0.057
NMR	10.0 (4.4)	8.3 (5.3)	0.255	9.8 (4.8)	0.868
Hemoglobin (g/L) (SD)	12.1 (2.0)	11.5 (2.5)	0.416	12.5 (2.9)	0.679
CRP (mg/dl) (SD)	12.2 (29.0)	34.2 (40.3)	0.043	37.1 (39.0)	0.037
ESR (mm/h) (SD)	18.2 (30.4)	23.3 (21.0)	0.465	18.8 (17.5)	0.942
Albumin (g/L) (SD)	37.3 (5.2)	33.6 (7.1)	0.054	35.7 (6.0)	0.401
Fibrinogen (g/L) (SD)	3.2 (1.1)	3.8 (1.7)	0.170	4.0 (1.0)	0.035

UC, ulcerative colitis; CDI, *Clostridium difficile* infection; SD, standard deviation; BMI, body mass index; WBC, white blood cells; NLR, neutrophil-to-lymphocyte ratio; LMR, lymphocyte-to-monocyte ratio; NMR, neutrophil-to-monocyte ratio; CRP, C-reactive protein; ESR, erythrocyte sedimentation rate.

**Table 3:** Demographic and basic laboratory values of Crohn's disease patients.

Variables	CD without CDI, inactive	CD without CDI, active	^*∗*^ *P* value	CD with CDI	^*∗*^ *P* value
Total number, *n* (%)	85 (60.3)	44 (31.2)		12 (8.5)	
Epidemiology					
Age (years), median (range)	32.0 (15–62)	29.0 (15–59)	0.86	30.0 (21–54)	0.926
Male gender	57 (67.1)	25 (56.8)	0.252	7 (58.3)	0.550
BMI (kg/m^2^) (SD)	18.7 (2.6)	18.4 (2.7)	0.546	18.7 (1.9)	0.995
Laboratory examination					
WBC (10^9^/L) (SD)	6.5 (2.1)	8.7 (3.2)	≤0.001	6.9 (2.5)	0.521
Neutrophil (10^9^/L) (SD)	4.5 (1.7)	6.3 (2.8)	≤0.001	4.8 (2.1)	0.637
Lymphocyte (10^9^/L) (SD)	1.3 (0.5)	1.3 (0.6)	0.406	1.4 (0.7)	0.454
Monocyte (10^9^/L) (SD)	0.5 (0.3)	0.7 (0.3)	≤0.001	0.5 (0.2)	0.748
NLR	4.0 (2.0)	5.5 (4.5)	0.010	4.1 (2.8)	0.928
LMR	3.1 (1.9)	2.3 (1.2)	0.009	3.2 (2.0)	0.821
NMR	10.6 (6.0)	11.1 (9.2)	0.709	10.7 (6.2)	0.974
Hemoglobin (g/L) (SD)	11.9 (1.9)	11.1 (2.3)	0.047	12.1 (1.2)	0.751
CRP (mg/dl) (SD)	19.3 (21.8)	47.4 (47.2)	≤0.001	35.6 (22.8)	0.018
ESR (mm/h) (SD)	21.4 (16.7)	33.0 (25.1)	0.002	20.3 (18.9)	0.828
Albumin (g/L) (SD)	33.6 (7.2)	32.1 (6.7)	0.262	35.2 (6.5)	0.452
Fibrinogen (g/L) (SD)	4.3 (1.2)	4.8 (1.3)	0.018	4.1 (1.0)	0.605

CD, Crohn's disease; CDI, *Clostridium difficile* infection; SD, standard deviation; BMI, body mass index; WBC, white blood cells; NLR, neutrophil-to-lymphocyte ratio; LMR, lymphocyte-to-monocyte ratio; NMR, neutrophil-to-monocyte ratio; CRP, C-reactive protein; ESR, erythrocyte sedimentation rate.

## Data Availability

The excel data used to support the findings of this study are available from the corresponding author upon request.

## References

[1] Molodecky N. A., Soon I. S., Rabi D. M. (2012). Increasing incidence and prevalence of the inflammatory bowel diseases with time, based on systematic review. *Gastroenterology*.

[2] Xavier R. J., Podolsky D. K. (2007). Unravelling the pathogenesis of inflammatory bowel disease. *Nature*.

[3] Jeong D. Y., Kim S., Son M. J. (2019). Induction and maintenance treatment of inflammatory bowel disease: a comprehensive review. *Autoimmunity Reviews*.

[4] Park J. H., Peyrin-Biroulet L., Eisenhut M., Shin J. I. (2017). IBD immunopathogenesis: a comprehensive review of inflammatory molecules. *Autoimmunity Reviews*.

[5] Walsh A. J., Bryant R. V., Travis S. P. L. (2016). Current best practice for disease activity assessment in IBD. *Nature Reviews Gastroenterology & Hepatology*.

[6] Reinisch W., Colombel J.-F., Sandborn W. J. (2015). Factors associated with short- and long-term outcomes of therapy for Crohn’s disease. *Clinical Gastroenterology and Hepatology*.

[7] Yoon J. Y., Park S. J., Hong S. P., Kim T. I., Kim W. H., Cheon J. H. (2014). Correlations of C-reactive protein levels and erythrocyte sedimentation rates with endoscopic activity indices in patients with ulcerative colitis. *Digestive Diseases and Sciences*.

[8] Henriksen M., Jahnsen J., Lygren I. (2008). C-reactive protein: a predictive factor and marker of inflammation in inflammatory bowel disease. Results from a prospective population-based study. *Gut*.

[9] Lindholm C. R., Siegel C. A. (2019). Are we ready to include prognostic factors in inflammatory bowel disease trials?. *Current Pharmaceutical Design*.

[10] Lester S. E., Proudman S. M., Lee A. T. Y. (2009). Treatment-induced stable, moderate reduction in blood cell counts correlate to disease control in early rheumatoid arthritis. *Internal Medicine Journal*.

[11] Wang Y., Fuentes H. E., Attar B. M., Jaiswal P., Demetria M. (2017). Evaluation of the prognostic value of neutrophil to lymphocyte ratio in patients with hypertriglyceridemia-induced acute pancreatitis. *Pancreatology*.

[12] Choi Y. H., Lee J. W., Lee S. H. (2019). A high monocyte-to-lymphocyte ratio predicts poor prognosis in patients with advanced gallbladder cancer receiving chemotherapy. *Cancer Epidemiology Biomarkers & Prevention*.

[13] He C., Zhang Y., Cai Z., Lin X. (2019). The prognostic and predictive value of the combination of the neutrophil-to-lymphocyte ratio and the platelet-to-lymphocyte ratio in patients with hepatocellular carcinoma who receive transarterial chemoembolization therapy. *Cancer Management and Research*.

[14] Torun S., Tunc B. D., Suvak B. (2012). Assessment of neutrophil-lymphocyte ratio in ulcerative colitis: a promising marker in predicting disease severity. *Clinics and Research in Hepatology and Gastroenterology*.

[15] Gao S. Q., Huang L. D., Dai R. J., Chen D. D., Hu W. J., Shan Y. F. (2015). Neutrophil-lymphocyte ratio: a controversial marker in predicting Crohn’s disease severity. *International Journal of Clinical and Experimental Pathology*.

[16] Cherfane C. E., Gessel L., Cirillo D., Zimmerman M. B., Polyak S. (2015). Monocytosis and a low lymphocyte to monocyte ratio are effective biomarkers of ulcerative colitis disease activity. *Inflammatory Bowel Diseases*.

[17] Truelove S. C., Witts L. J. (1955). Cortisone in ulcerative colitis. *Bmj*.

[18] Schroeder K. W., Tremaine W. J., Ilstrup D. M. (1987). Coated oral 5-aminosalicylic acid therapy for mildly to moderately active ulcerative colitis. *New England Journal of Medicine*.

[19] Elliott P. R., Lennard-Jones J. E., Hathway N. (1980). Simple index of Crohn’s disease activity. *The Lancet*.

[20] Cerrillo E., Moret I., Iborra M. (2019). A nomogram combining fecal calprotectin levels and plasma cytokine profiles for individual prediction of postoperative crohn’s disease recurrence. *Inflammatory Bowel Diseases*.

[21] Manceau H., Chicha-Cattoir V., Puy H., Peoc’h K. (2017). Fecal calprotectin in inflammatory bowel diseases: update and perspectives. *Clinical Chemistry and Laboratory Medicine*.

[22] Khan K., Schwarzenberg S. J., Sharp H., Greenwood D., Weisdorf-Schindele S. (2002). Role of serology and routine laboratory tests in childhood inflammatory bowel disease. *Inflammatory Bowel Diseases*.

[23] Beyazit Y., Koklu S., Tas A. (2012). Serum adenosine deaminase activity as a predictor of disease severity in ulcerative colitis. *Journal of Crohn’s and Colitis*.

[24] Zezos P., Papaioannou G., Nikolaidis N. (2009). Elevated markers of thrombin generation and fibrinolysis in patients with active and quiescent ulcerative colitis. *Med Sci Monit*.

[25] Kayapinar O., Ozde C., Kaya A. (2019). Relationship between the reciprocal change in inflammation-related biomarkers (Fibrinogen-to-Albumin and hsCRP-to-Albumin ratios) and the presence and severity of coronary slow flow. *Clinical and Applied Thrombosis/Hemostasis*.

[26] Don B. R., Kaysen G. (2004). Poor nutritional status and inflammation: serum albumin: relationship to inflammation and nutrition. *Seminars in Dialysis*.

[27] Selby W. S., Janossy G., Bofill M., Jewell D. P. (1984). Intestinal lymphocyte subpopulations in inflammatory bowel disease: an analysis by immunohistological and cell isolation techniques. *Gut*.

[28] Zahorec R. (2001). Ratio of neutrophil to lymphocyte counts--rapid and simple parameter of systemic inflammation and stress in critically ill. *Bratislavske lekarske listy*.

[29] Celikbilek M., Dogan S., Ozbakır O. (2013). Neutrophil-lymphocyte ratio as a predictor of disease severity in ulcerative colitis. *Journal of Clinical Laboratory Analysis*.

[30] Shi C., Pamer E. G. (2011). Monocyte recruitment during infection and inflammation. *Nature Reviews Immunology*.

[31] Mee A. S., Berney J., Jewell D. P. (1980). Monocytes in inflammatory bowel disease: absolute monocyte counts. *Journal of Clinical Pathology*.

[32] Jones G. R., Bain C. C., Fenton T. M. (2018). Dynamics of colon monocyte and macrophage activation during colitis. *Frontiers in Immunology*.

